# Uncommon Etiologies of Acute Abdominal Pain: A Case Report on Omental Infarction

**DOI:** 10.7759/cureus.62493

**Published:** 2024-06-16

**Authors:** Yesenia Brito, Hadeel Assi, Ana I Gonzalez, Salsabela Shaban, Frederick Tiesenga, Juaquito Jorge

**Affiliations:** 1 Surgery, St. George's University School of Medicine, True Blue, GRD; 2 Medicine, St. George's University School of Medicine, True Blue, GRD; 3 Surgey, St. George's University School of Medicine, True Blue, GRD; 4 General Surgery, West Suburban Medical Center, Chicago, USA; 5 General and Bariatric Surgery, Tiesenga Surgical Associates, Elmwood Park, USA

**Keywords:** acute abdominal emergencies, rare cause of acute abdominal pain, hypercoagulation, acute colitis, epiploic appendagitis, omental torsion, omental infarction

## Abstract

Omental infarction is an uncommon cause of abdominal pain. The condition is often misdiagnosed due to its clinical similarity to more common abdominal pathologies like appendicitis and cholecystitis. This report presents the case of a 57-year-old female with a one-week history of left-sided abdominal pain, initially aggravated by eating and defecation. The patient, a long-term smoker with a complex medical history that includes deep vein thrombosis and pulmonary embolism, was hemodynamically stable on presentation. A CT scan revealed a nodular infiltration consistent with an omental infarct. Conservative management was pursued, resulting in symptom resolution by the third day of hospitalization.

This case underscores the diagnostic challenges associated with omental infarction, particularly its differentiation from other causes of acute abdominal pain. It highlights the importance of considering rare etiologies in patients with atypical presentations and emphasizes the role of imaging, particularly CT scans, in accurate diagnosis. The patient's successful conservative management aligns with current recommendations, which advocate for non-surgical treatment in most cases. This approach avoids unnecessary surgical interventions and ensures a favorable prognosis with low complication rates in patients with prompt and appropriate management.

## Introduction

Abdominal pain accounts for 4-5% of emergency room (ER) visits [1]. The initial step in finding the culprit of abdominal pain is to complete a full history and physical examination of the patient. Identifying the onset, location, duration, intensity, and predisposing factors to abdominal pain helps narrow down the differential diagnoses and minimize unnecessary testing. When the patient is unable to provide an extensive history due to acuity, the location of the pain can aid in determining the next best step of management. Generalized abdominal pain can be associated with bowel ischemia, pancreatitis, and obstruction, prompting the need for imaging [1]. The right upper abdominal quadrant can be affected by gallbladder pathologies, colitis, and myocardial infarction, while left upper abdominal quadrant tenderness has been documented in myocardial infarction and pancreatitis [1]. Abdominal pain associated with guarding will warrant a surgical consult along with imaging and laboratory testing [1]. In cases where myocardial infarction is suspected, ECGs should be considered as part of patient management [1].

Although algorithms exist to diagnose and manage abdominal pain, rare etiologies can often be overlooked due to their low incidence and similarity with common ones [2]. Ischemic colitis, for example, has an incidence rate of 16.3 cases per 100,000 individuals and is associated with aging, atherosclerotic disease, increased adipose abdominal tissue, and smoking [3]. The presentation of ischemic colitis can vary depending on the affected colonic segment and is often confused with other disease processes [3]. Epiploic appendagitis is another infrequently seen cause of abdominal pain that mimics diverticulitis and appendicitis [4]. Although primary epiploic appendagitis will often resolve without the need for medical intervention, the presentation could lead to invasive and unwarranted surgical procedures [4].

Adding to the list of rare causes of abdominal pain, omental infarction has an incidence of less than 1% [5], with 90% of the cases involving the right side due to increased omental mobility [2]. The greater omentum consists of fatty tissue, blood vessels, and the visceral peritoneal layers of the stomach and transverse colon [2,6]. It was once thought to be a provider of insulation, but now we know that it has immunologic properties, systemic vascular components, and even a central nervous system connection [6]. Omental infarction can present with acute abdominal pain, nausea, vomiting, and less frequently with fever [2,7]. It is classified as a primary or secondary infarction [5]. Primary omental infarctions are those of unknown origin, termed idiopathic, while secondary infarctions are generated by a condition or physical state [8]. Although the exact cause of omental infarction is usually unknown [2], the two most commonly identifiable etiologies include vascular torsion and thrombosis secondary to a hypercoagulable state [7,8]. For example, COVID-19 infection and the COVID-19 vaccine are linked to hypercoagulability and direct viral effects on the gastrointestinal tract [7-9]. Although to date a link between the COVID-19 vaccine and omental infarction has not been reported, multiple cases of omental infarction have been reported in COVID-19-positive patients, and it is believed to be due to a higher propensity toward arterial thromboembolism in this patient population [8,9]. Other causes included hernias, trauma, and neoplasms [5]. Obesity is a known risk factor for the development of omental infarction, but it does not correlate with condition severity [5]. Other risk factors include being a male between 40 and 50 years of age [2].

Diagnosis of omental infarct can be challenging as it mimics appendicitis and cholecystitis [2], which are more commonly seen causes of right-sided abdominal pain. In most cases, the use of a computed tomography (CT) scan is the first step in diagnosis and is considered the gold standard [2,7,8]. However, the use of ultrasound (US) has been documented in cases where the patient is relatively stable and whose management does not warrant prompt surgical intervention [2]. Typical CT findings include an oval-shaped region of fat attenuation with surrounding soft tissue and local inflammation [10]. On US, signs of omental infarct include echogenic fatty tissue lacking internal vascularity [10]. The incidence of omental infarction in patients with acute abdominal pain is 0.3% [5], which makes the condition quite rare.

Mild omental infarction can be managed in an outpatient setting, and hospitalization is warranted for those with moderate or severe cases [5]. Treatment can be divided into conservative or surgical management, with conservative treatment being the most commonly used and encompassing a success rate of over 80% in those that warrant it [5,7]. Conservative management consists of analgesics, antispasmodics, and oral or parenteral antibiotics [5]. Surgical management is reserved for patients who fail conservative management or have a severe presenting illness [5,7]; however, data suggest that the mean length of hospitalization is significantly reduced in those with initial surgical management from 5.1 days to 2.5 days [7]. Factors associated with failure of conservative treatment include a white blood cell count of over 12,000 ml and a mean age of 37.9 years [7]. Patients who undergo conservative management will require a three-month follow-up period after symptomatic resolution to monitor for abscess formation [7]. Surgically managed cases do not require post-surgical follow-up [7].

Overall, omental infarct is a rare condition associated with multiple etiologies. The course of the disease is heavily dictated by its management and severity. There is a relatively good prognosis and low risk of complications in promptly stabilized patients with omental infarct. It is important to highlight the challenges faced during clinical diagnosis, as physical presentation could mimic other more commonly seen pathologies.

## Case presentation

A 57-year-old female presented to the ER with a one-week history of left-sided abdominal pain. The pain was aggravated by eating and defecation. She reported associated symptoms of non-bloody diarrhea, describing the stool as dark-colored. Additionally, the patient experienced some dysuria but denied hematuria or increased urinary frequency. She also denied fever, chills, nausea, vomiting, recent travel, or antibiotic use. It is noteworthy that the patient has been a daily smoker, consuming 1 pack per day for the past 40 years.

The patient’s past medical history was significant for asthma, COPD, diabetes mellitus, hypertension, hypothyroidism, anxiety, depression, seizure, deep vein thrombosis (DVT), and pulmonary embolism (last year, currently on rivaroxaban). Her most recent colonoscopy on April 25, 2022, revealed a 7 mm adenomatous polyp in the rectum and no signs of diverticula or diverticulitis. She also has a surgical history that includes cholecystectomy, a ventral hernia repair with mesh in July 2020, and a thrombectomy in March 2023, in which she reported that “clots were removed from my heart and lungs.” The patient received four doses of the Pfizer COVID-19 vaccine on 03/28/2021, 04/18/2021, 02/14/2022, and 08/12/2022. 

**Figure 1:**
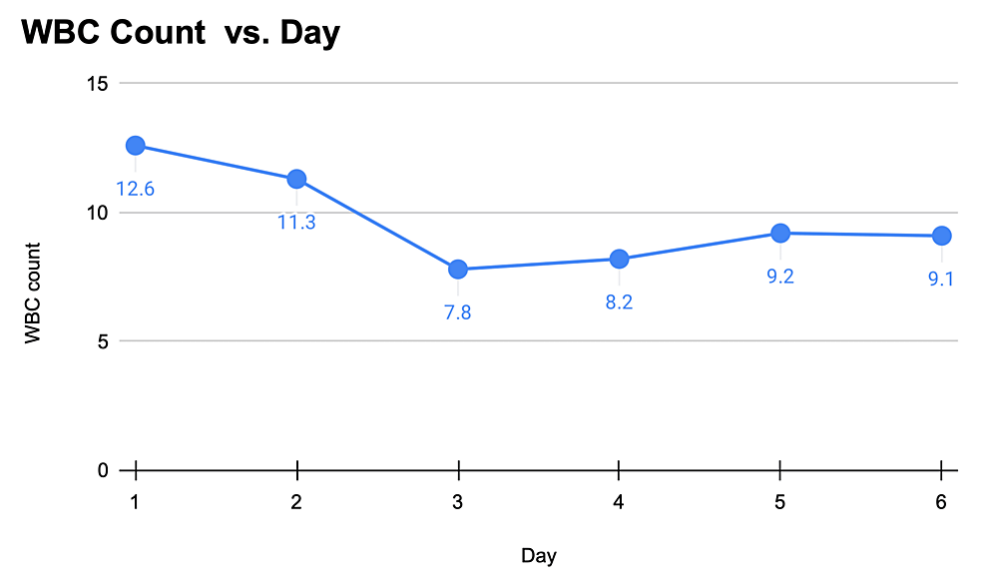
WBC count in microliters vs. duration of hospital stay per day

**Figure 2:**
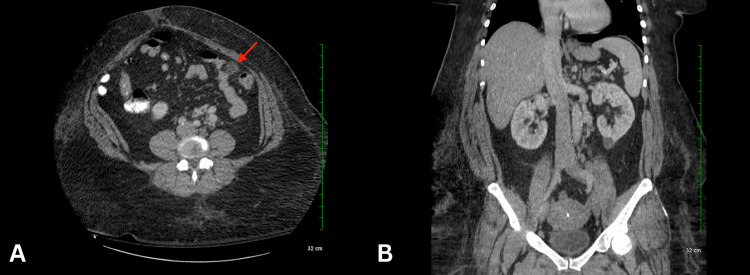
(A) There is infiltration of the omentum in the left mid abdomen just adjacent to the descending colon (red arrow). This may represent an area of omental infarct or possibly focal epiploic appendagitis. (B) The gallbladder is surgically absent. No intra- or extrahepatic biliary ductal dilatation. The appendix was not visualized, although there was no pericecal inflammation.

On physical examination, the patient was hemodynamically stable. The vital signs were within normal limits. Her body mass index was 52.94 kg/m². Upon abdominal palpation, there was tenderness throughout the abdomen, more significant in the left lower quadrant. No distension, rebound, or guarding was present. Laboratory exams showed leukocytosis (Figure 1). Stool studies were negative for occult blood and *Clostridium difficile*. A CT scan of the abdomen showed a 2.2 cm nodular infiltration in the left anterior abdomen, possibly an omental infarct or, less likely, epiploic appendagitis (Figure 2).

Upon consultation with the general surgery team, conservative management was recommended, with surgical intervention reserved only for the deterioration of the patient's condition. Additionally, the gastroenterology team noted that the patient's diarrhea and leukocytosis suggested acute colitis, which could be infectious or ischemic. Subsequently, the patient was admitted for observation and underwent conservative management, including analgesics, IV antibiotics, IV fluids, and rest. By the fifth day of admission, her diarrhea and abdominal pain had resolved, leading to her discharge. A follow-up abdominal CT scan was advised within the next three months to monitor her condition.

## Discussion

Omental infarction, epiploic appendagitis, and acute colitis can have overlapping causes and presentations. There are several causes of omental infarction, torsion being a significant one. Primary omental torsion is often related to congenital factors, such as abnormalities in omental attachment or a bifid omentum. Additionally, congenital conditions that predispose a patient to hypercoagulable states can lead to venous thrombosis, further contributing to the risk of omental infarction [11]. However, secondary torsion is an acquired condition, often resulting from factors such as malignancy or scar tissue [11]. Predisposing factors include obesity, surgery, laxative use, sudden changes in posture, congestive heart failure, and superior mesenteric artery occlusion [11]. Abdominal pain is typically located on the right side (90% of cases), as the omentum is more mobile and longer on that side [2,11]. The patient in this case presented with left-sided pain, which is only seen in 10% of cases of omental infarct [2].

The patient in this case had multiple predisposing factors for omental infarction, all of which can lead to hypercoagulable states. These factors include a history of ventral hernia, obesity, smoking, ventral hernia repair, DVT, pulmonary embolism, and COVID-19 vaccination. She received her last COVID-19 vaccine dose on 08/12/2022, preceding the hypercoagulable events of pulmonary embolism and DVT. As previously noted, COVID-19 vaccines have been associated with hypercoagulable states [7-9], a known risk factor for omental infarction. While cases of omental infarction following the COVID-19 vaccine have not been reported in the literature, it is important to mention that a propensity toward hypercoagulability exists and could be potentially linked to omental infarct. 

Like omental infarction, the causes of epiploic appendagitis can be classified into primary and secondary [12]. Primary epiploic appendagitis is thought to be a sterile inflammatory condition caused by the torsion of a single appendage [12]. This torsion can lead to ischemia and infarction, resulting in aseptic fat necrosis and spontaneous venous thrombosis [12]. Secondary epiploic appendagitis is when a healthy epiploic appendage is affected by another disease state, such as bacterial infection of an adjacent organ or inflammatory bowel disease, leading to epiploic appendagitis [12]. The most reported cause of secondary epiploic appendagitis is diverticulitis [12]. 

Ischemic colitis is typically caused by an unidentified nonocclusive insult to the small vessels supplying the colon, but specific etiologies should be considered, especially in younger patients [13]. Potential causes include drugs, long-distance running, and hypercoagulable state, with Factor V Leiden mutation being the most common [13]. It is important to review medications associated with ischemic colitis, such as non-steroidal anti-inflammatory drugs (NSAIDs), vasoconstrictors, and diuretics, in all patients [13]. Systemic illnesses like sepsis or heart failure can lead to transient colonic hypoperfusion and resultant ischemia, often with a worse prognosis due to the severity of the underlying condition [13]. Additionally, infections, vascular and bowel surgeries, and less common factors such as vasculitis and trauma can also cause ischemic colitis [13]. 

Omental infarction, epiploic appendagitis, and ischemic colitis can present with similar symptoms, including nausea, vomiting, anorexia, diarrhea, and fever [11-13]. Patients with omental infarction usually present acutely with localized right lower quadrant pain [11]. In contrast, epiploic appendagitis often presents with early satiety and moderate weight loss, with abdominal pain that is usually subacute, recurrent, dull, and constant, localized to the left abdomen in 60-80% of cases [12]. Physical examination typically reveals localized tenderness [12]. On the other hand, the pain in ischemic colitis is usually crampy, varies in location, and is associated with bloody diarrhea. 

The gold standard imaging modality for diagnosing and differentiating between omental infarction, epiploic appendagitis, and ischemic colitis is the CT scan [11]. In omental infarction, we expect a triangular or oval heterogeneous fatty mass between the anterior abdominal wall and the transverse or ascending colon [11]. Omental torsion, on the other hand, will show a whirled pattern of concentric linear strands [11]. The size of omental infarction on CT varies depending on many variables, like age [11]. Typically, an omental infarction lesion on CT is larger than that of epiploic appendagitis; omental infarction appears cake-like and is centered in the omentum, and is located medial to the cecum or ascending colon [12].

CT is particularly effective for identifying epiploic appendagitis, which presents as an approximately 3 cm, oval-shaped, fat-dense paracolic lesion with a hyperattenuating ring indicating the inflamed visceral peritoneum [12]. In cases of ischemic colitis, CT findings include segmental thickening of the colon and "thumbprinting," which results from sub-epithelial hemorrhage [13]. While other imaging modalities can be used for diagnosing these conditions, the focus here is on distinguishing their presentation on CT scans.

The patient's abdominal CT revealed diverticulosis without evidence of diverticulitis. While the symptoms and imaging findings showed some overlap between omental infarction and epiploic appendagitis, her presentation inclined toward omental infarction as the definitive diagnosis due to acuity, course, characteristics, and prompt response to conservative management. The abdominal CT scan showed a 2.2 cm nodular infiltration in the left anterior abdomen, consistent with the shape of an omental infarction. It is important to highlight that epiploic appendagitis is known to present as a lesion with a ring, which would be inconsistent with our radiologic findings. Moreover, this patient did not have torsion or a bacterial infection, making epiploic appendagitis a less favorable diagnosis. Omental infarction and epiploic appendagitis generally have good prognosis but can occasionally present with complications. Some of the complications of omental infarction are adhesions and intra-abdominal abscesses, necessitating close monitoring of the patient in the first few days [14]. For follow-up, a US should be performed initially, followed by annual CT scans for the next three years [14]. Some of the complications of epiploic appendagitis include local abscess, peritonitis, bowel obstruction, and intussusception [12]. These complications mainly arise due to adhesions [12]. Moreover, when an appendage becomes necrotic, and the inflammation subsides, nonviable appendages get absorbed by the body, leading to detachment. This detachment can lead to the loosening of intraperitoneal bodies, which may cause intestinal obstruction or urine retention [12].

In the past, all three conditions were diagnosed and treated surgically. However, the current recommendation is to diagnose them using a CT scan and manage them conservatively when possible [12]. Authors recommend conservative management for omental infarction when diagnosed radiologically [12]. Even though conservative management has shown decreased effectiveness in patients with WBC above 12,000 and patients older than 37.9 years of age [7], this patient was successfully treated conservatively, using IV antibiotics, IV fluid, and analgesics. 

Epiploic appendagitis is mostly self-limiting and managed with NSAIDs for symptomatic relief [12]. Nevertheless, some patients with epiploic appendagitis but without sepsis are treated successfully with antibiotics, such as ciprofloxacin, and anti-inflammatory drugs, such as ibuprofen, at the physician's discretion [12]. With conservative management, both omental infarction and epiploic appendagitis can resolve within a few days to a few weeks [12]. Similarly, conservative management is also the preferred approach for treating ischemic colitis [13]. This therapy consists of IV fluids, bowel rest, bowel decompression, avoiding vasopressor medications, and cessation of any other potentially offending drugs [13]. However, if symptoms persist, surgical interventions, such as laparoscopy, may be required for all three conditions [12-14].

Our patient’s medical history and comorbidities undoubtedly played a significant role in shaping the presentation and management of the omental infarction. The recent past medical history of pulmonary embolism and DVT indicates the presence of an underlying hypercoagulable state predisposing the patient to thrombotic events, including omental infarction. Furthermore, the multiple COVID-19 vaccines and the presence of multiple comorbidities, such as diabetes mellitus and hypertension, may have contributed to vascular compromise and increased susceptibility to ischemic events. The interplay between these comorbidities and the pathophysiology of omental infarction underscores the complexity of managing such cases. It emphasizes the importance of thoroughly understanding the patient's medical history in guiding diagnostic and therapeutic interventions.

## Conclusions

Omental infarction presents a diagnostic challenge due to its rarity and resemblance to more common abdominal pathologies. However, prompt recognition and accurate diagnosis are crucial to avoid unnecessary surgical interventions and ensure optimal patient outcomes. This case illustrates the importance of a comprehensive history, physical examination, and appropriate imaging in evaluating patients with acute abdominal pain.

Conservative management, as demonstrated in this case, is often effective in resolving symptoms and avoiding surgical interventions. This approach aligns with current recommendations and underscores the significance of individualized patient care based on clinical presentation and imaging findings. Continued vigilance and awareness of rare abdominal pathologies, such as omental infarction, are essential for clinicians to provide timely and appropriate management, ultimately leading to favorable patient outcomes. Further studies and case reports are warranted to enhance our understanding of this rare condition and optimize its management strategies.
